# Feeding Bugs to Bugs: Edible Insects Modify the Human Gut Microbiome in an *in vitro* Fermentation Model

**DOI:** 10.3389/fmicb.2020.01763

**Published:** 2020-07-23

**Authors:** Wayne Young, Sai Krishna Arojju, Mark R. McNeill, Elizabeth Rettedal, Jessica Gathercole, Nigel Bell, Penny Payne

**Affiliations:** ^1^Food Nutrition & Health Team, AgResearch Grasslands, Palmerston North, New Zealand; ^2^Riddet Institute, Massey University, Palmerston North, New Zealand; ^3^High-Value Nutrition, National Science Challenges, Auckland, New Zealand; ^4^Forage Genetics Team, AgResearch Grasslands, Palmerston North, New Zealand; ^5^Biocontrol and Biosecurity Team, AgResearch Lincoln, Christchurch, New Zealand; ^6^Proteins and Metabolites Team, AgResearch Lincoln, Christchurch, New Zealand; ^7^Soil Biology Team, AgResearch Ruakura, Hamilton, New Zealand; ^8^People and Agriculture Team, AgResearch Ruakura, Hamilton, New Zealand

**Keywords:** insects, microbiota, digestion, fecal culture, gut, prebiotics, novel foods

## Abstract

We here report a study characterizing the potential for edible insects to act as a prebiotic by altering the bacterial composition of the human fecal microbiome, using batch cultures inoculated with fecal adult human donors. Black field cricket nymphs, grass grub larvae, and wax moth larvae were subjected to an *in vitro* digestion to simulate the oral, gastric, and small intestinal stages of digestion. The digested material was then dialyzed to remove small molecules such as amino acids and free sugars to simulate removal of nutrients through upper gastrointestinal tract digestion. The retentate, representing the digestion resistant constituents, was then fermented in fecal batch cultures for 4, 7, and 15 h to represent rapid and longer fermentation times. Batch cultures without any added substrates were also set up to act as controls. Additionally, phosphate-buffered saline was used as a no-protein control and milk powder as “standard” protein control. At the end of the incubation period, the bacterial pellets were collected for microbiome analysis by 16S rRNA gene amplicon sequencing. Analysis of fecal cultures showed striking differences in community composition. Each substrate led to significant differences across a wide range of taxa compared to each other and PBS controls. Among the differences observed, digested grass grub larvae increased proportions of *Faecalibacterium* and the *Prevotella* 2 group. Black field crickets increased the prevalence of the *Escherichia*–*Shigella* group, *Dialister* genus, and a group of unclassified *Lachnospiraceae*. Wax moth larvae promoted the expansion of the same group of unclassified *Lachnospiraceae* and the *Escherichia*/*Shigella* group. The increased *Faecalibacterium* observed in the cultures with grass grub larvae represents a noteworthy finding as this bacterium is widely thought to be beneficial in nature, with demonstrated anti-inflammatory properties and associations with gut health. We conclude that insects can differentially modulate the microbiome composition in batch cultures inoculated with adult fecal material after simulated *in vitro* digestion. Although the physiological impact *in vivo* remains to be determined, this study provides sound scientific evidence that investigating the potential for consuming insects for gut health is warranted.

## Introduction

The benefits of insect consumption are well documented in the literature (Patel et al., 2019). Relative to livestock, insects are a more sustainable and efficient food source, requiring minimum water and space (van Huis et al., 2013; Deroy et al., 2015). The consumption of insects for food is a traditional practice in many human societies, especially in Asia and Africa, and common in low-income groups in these countries. By contrast, in most Western countries people view entomophagy with disgust or even as culturally inappropriate therefore consumption is infrequent (van Huis et al., 2013). However, with greater awareness of the environmental footprint associated with the livestock industry and concerns around sustainability of agriculture and the impacts of climate change on productive systems, there is becoming a greater recognition and acceptance of insects as an additional and healthy protein source (Bessa et al., 2017; Loveday, 2019; Patel et al., 2019). Insects are composed of 30–80% protein on a dry matter basis (Rumpold and Schlüter, 2013; Patel et al., 2019) and are far more efficient in converting feed to bodyweight than traditional mammalian livestock (van Huis and Oonincx, 2017).

Insects may also possess other health promoting properties beyond provision of macronutrients. Chitin, a polymer of N-acetylglucosamine, is the primary constituent of the exoskeleton of insects and is also resistant to mammalian digestive enzymes. Therefore, chitin has the potential to reach the large bowel intact, where it could act as a prebiotic to promote the growth of beneficial members of the gut microbiome (Borrelli et al., 2017). This effect has recently been demonstrated in humans where consumption of edible crickets by healthy volunteers increased the relative abundance of fecal *Bifidobacterium* (Stull et al., 2018), a genera typically associated with beneficial properties (O’Callaghan and van Sinderen, 2016). However, insects as a class (Insecta) are highly diverse; some estimates place over half of all non-microbial biodiversity on Earth as insects (Larsen et al., 2017; Stork, 2018). Associated with this biodiversity, it is conceivable that different species of edible insects may have different effects on the human gut microbiota. To test this hypothesis, we explored the potential of a range of New Zealand domiciled insects for modulating the human gut microbiome using an *in vitro* batch fermentation model to simulate colonic fermentation. To better mimic the human digestive system, the insects were first subjected to an *in vitro* digestion, followed by removal of small molecules by dialysis, before fecal fermentation.

## Materials and Methods

### Insect Collection

Late instar nymphs and adult black field crickets (*Teleogryllus commodus*) (Orthoptera: Gryllidae) were collected from beef-grazed grassland on a lifestyle farm near Te Mata in the Waikato region, New Zealand. Grass grub larvae (*Costelytra giveni*) (Coleoptera: Scarabeidae) were collected from a ryegrass (*Lolium perenne* L./white clover (*Trifolium repens* L) pasture on the Lincoln University Research Dairy Farm at Lincoln, Canterbury, New Zealand. The black field crickets and grass grubs were shipped overnight to the AgResearch laboratory in Palmerston North, New Zealand and euthanized by freezing. Greater wax moth larvae (*Galleria mellonella* L.) (Lepidoptera: Pyralidae) were purchased from Biosuppliers (Auckland, New Zealand^1^) and similarly euthanized. Black field cricket is a native species, grass grub an endemic species and greater wax moth an exotic species.

### *In vitro* Digestion

To mimic the human digestive process, the insects were subjected to *in vitro* digestion as described previously (Minekus et al., 2014) with the following modifications.

Each type of insect were ground while still frozen into a paste using a mortar and pestle. Five grams of each type of ground insect was added to separate tubes followed by the addition of 5 mL of simulated salivary fluid [SSF; 15.1 mM KCl, 3.7 mM KH_2_PO_4_, 13.6 mM NaHCO_3_, 0.15 mM MgCl_2_(H_2_O)_6_, 0.06 mM (NH_4_)_2_CO_3_, pH 7] and 1500 U/mL of salivary α-amylase (Sigma A6380, E.C. 3.2.1.1; St. Louis, Mo, United States). The mixtures were then incubated at 37°C for 2 min.

After incubation, 7 mL of simulated gastric fluid (SGF; 6.9 mM KCl, 0.9 mM KH_2_PO_4_, 25 mM NaHCO_3_, 47.2 mM NaCl, 0.1 mM MgCl_2_(H_2_O)_6_, 0.5 mM (NH_4_)_2_CO_3_, pH 3) was added to each sample mixture, followed by 1 mL of pepsin stock solution (2000 U/mL in SGF) (Sigma P6887). This was followed by the addition of 100 μL of 300 mmol L^−1^ CaCl_2_, 1 mL of lipase solution (800 U/mL [Sigma L0382] in SGF), and water to make a total volume of 20 mL. The samples were then incubated at 37°C for 2 h with shaking. To mimic the small intestinal phase of digestion, 11 ml of simulated intestinal fluid (SIF; 6.8 mM KCl, 0.8 mM KH_2_PO_4_, 85 mM NaHCO_3_, 38.4 mM NaCl, 0.33 mM MgCl_2_(H_2_O)_6_, pH 6.5), 2.5 mL of bile salt solution (16 mM bile salt [Sigma B8756] in SIF, pH 7), 40 μL of 300 mM CaCl_2_, and 1.13 mL water was added to the resulting chyme. This was then incubated for 10 min at 37°C in a Ratek Orbital shaker (Ratek Instruments Pty Ltd., Boronia, Australia) set to 50 rpm. Following incubation, 5 mL of pancreatin solution (4.33 g of pancreatin powder [37452 FIP-U/mg, AppliChem A0585, Ottoweg, Darmstadt, Germany] in 10 mL of SIF) was added and the resulting solution incubated at 37°C for 2 h in a Ratek Orbital shaker set to 50 rpm. After incubation the enzymes were heat inactivated by microwaving on high for 1 min. The tubes were then cooled on ice and stored in the fridge overnight.

Following digestion, the samples were dialyzed using 24 cm of MWCO dialysis tubing (molecular weight cut-off 100–500, diameter 31 × 20 mm, 3.1 mL per cm) for 24 h with three water changes at regular intervals. This step was undertaken to mimic the removal of monosaccharides and small peptides released by the digestion process, which would be have been absorbed in the small intestine *in vivo*. The resulting retentate, representing the digestion resistant fraction, was diluted approximately 2.5-fold as a result of the dialysis step and then aliquoted into 50 ml tubes and frozen at −80°C. These aliquots, representing approximately 0.25 g of digested insect were used as a substrate in batch fecal cultures (mixed with the equivalent of 1 g of fresh feces) to simulate the fermentation of these substrates in the large bowel. As a positive substrate control 5 g of whole milk powder was subjected to the same *in vitro* digestion process described above, with PBS providing a negative protein control treatment.

### Fecal Collection and Fermentation

Fecal samples from three healthy adult donors (no exposure to antibiotics or probiotics within the last 3 months) were collected and stored overnight at 4°C before transport to the AgResearch Grasslands laboratory the next morning. The fecal samples were not stored in a preservative medium to avoid the addition of other potential substrates for microbial fermentation (De Weirdt et al., 2010). The fecal samples were pooled (12 g in total) and placed into a filter bag (filter size 0.28 mm; MicroScience Blender Bag SOR-207) with 60 mL of sterile degassed phosphate buffered saline (PBS). After massaging the bag to move the soluble fecal material through the filter, the fecal water was collected to inoculate the batch cultures.

Five mL of 200 mM (2×) potassium phosphate buffer (pH 7.2) was added to Hungate tubes and then the sample digest retentate solutions added to media at a 1:1 (w/v) ratio with the media degassed with nitrogen. 100 μL of 3% sterile filtered cysteine solution (Sigma C7352) was added and the tubes allowed to stand for 5 min before adding 2 mL of the fecal water based on previous literature (Edwards et al., 1996). Tubes were prepared in triplicate for each condition and time point and were all inoculated at the same time. The time zero samples were inoculated and then immediately transferred onto ice. Tubes were then incubated under static conditions at 37°C for 4, 7, and 15 h. These time points were chosen to represent times required to metabolise relatively easily digested (4 h) or more resistant substrates (15 h).

For collection, the sample was mixed by inverting the tube followed by vortexing. Two mL was transferred to a microfuge tube and centrifuged at 12,000 × *g* for 10 min at 4°C. The supernatant was decanted into separate tubes and immediately frozen at −80°C for future analyses. The remaining bacterial pellet was stored at −80°C for later DNA extraction.

### Microbiome Sequencing and Analysis

Metagenomic DNA was extracted from culture pellets using Macherey Nagel NucleoSpin Soil kits (Düren, Germany) following manufacturer’s instructions with the addition of a bead beating step using a BioSpec Mini-Beadbeater 96 (Bartlesville, OK, United States) set to 4 min. DNA samples were then analyzed by 16S rRNA gene amplicon sequencing using the Illumina MiSeq platform with 2 × 250 bp paired-end sequencing with PCR primers targeting the V3 and V4 region:

#### Forward Primer

5′-TCGTCGGCAGCGTCAGATGTGTATAAGAGACAGCCTA CGGGNGGCWGCAG.

#### Reverse Primer

5′-GTCTCGTGGGCTCGGAGATGTGTATAAGAGACAGGAC TACHVGGGTATCTAATCC.

PCR thermal cycler conditions were used as specified in the Illumina library preparation protocol (95°C for 3 min; 25 cycles of [95°C for 30 s, 55°C for 30 s, 72°C for 30 s]; 72°C for 5 min; Hold at 4°C (Illumina 2015).

Sequence reads were quality trimmed using the following parameters in Qiime 2 (Caporaso et al., 2010; Bolyen et al., 2018): Adapter sequences were removed using the cutadapt function, paired reads joined using vsearch with a minimum overlap of 20 bp, reads were quality trimmed with a 25 q-score cut off, remaining reads denoised and chimera checked using the deblur algorithm. Sequence reads were classified by aligning against the Silva 132 small subunit ribosomal RNA database. Alpha diversity was assessed using the Faith’s Phylogenetic Diversity, Chao1, Shannon, and observed OTUs indices, and beta diversity was compared using Principal Coordinate Analysis (PCoA) of weighted unifrac phylogenetic distances. The sampling depth used for alpha and beta diversity analysis was 13500 reads. Comparisons of overall community compositions were performed using permutational multivariate analysis of variance (PERMANOVA) using distance matrices as implemented in the adonis function in the vegan package (Dixon, 2003) for R. *Post hoc* pairwise permutation MANOVAs were analyzed using the RVAideMemoire package in R (Hervé, 2019). Dispersion between groups was tested using the betadisper function in vegan. Differences in the relative abundances of individual taxa at the genus level were analyzed by permutation two factor ANOVA (substrate × time) using the aovp function from the lmPerm package (Wheeler and Torchiano, 2016) for R. Resulting *P*-values were adjusted for multiple testing using the False Discovery Rate (FDR). *Post hoc* pair-wise analysis was performed using Fisher’s LSD test.

Sequences are publicly available from the NCBI Sequence Read Archive (SRA) under accession PRJNA566047.

## Results

Following quality trimming, denoising, and chimera removal, the median number of paired-end reads was 16878, with a minimum and maximum number of reads of 13591 and 18641, respectively.

**FIGURE 1:**
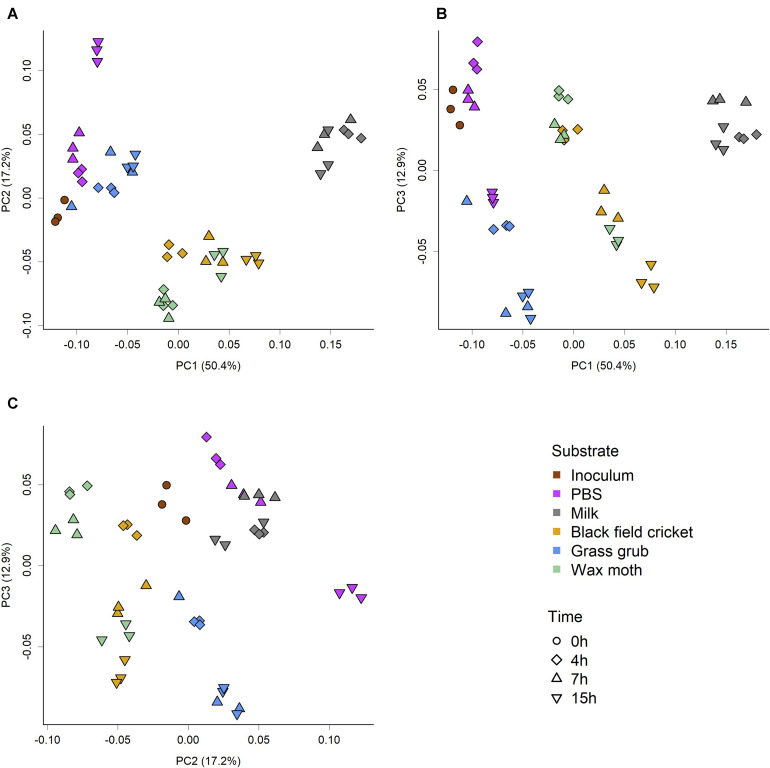
PCoA score plots of weighted Unifrac phylogenetic distances showing separation of microbiome community structure based on substrate and culture time. **(A)** PC1 vs PC2, **(B)** PC1 vs PC3, **(C)** PC2 vs PC3. PERMOVA (adonis) *P*-value of <0.001 indicates groups had significantly different compositions. Pairwise permutation MANOVAs showed all substrate were significantly different (*P* < 0.01), while 4 and 15 h cultures were significantly different to each other (*P* = 0.0.2). Analysis of group dispersion shown no significant differences in variation between groups (*P* = 0.15).

The overall community composition varied substantially between substrate and culture time, as indicated by the PCoA scores plot of weighted Unifrac phylogenetic distances (Figure 1). The significance of the observed separation between groups was confirmed by two-factor PERMANOVA (substrate × time), which showed a significant effect of substrate (*P* < 0.001, *R*^2^ = 0.76, *F* = 139.66), time (*P* < 0.001, *R*^2^ = 0.14, *F* = 106.12), and a significant interaction between substrate and time (*P* < 0.001, *R*^2^ = 0.05, *F* = 9.13). Pairwise permutation MANOVAs using the RVAideMemoire package in R showed all substrate were significantly different to each other (*P* < 0.01), while 4 h and 15 h cultures were also significantly different (*P* = 0.02). However, PERMANOVA showed the inoculum and PBS cultures were not significantly different (*P* = 0.09); see Tables 2–4.

**FIGURE 2:**
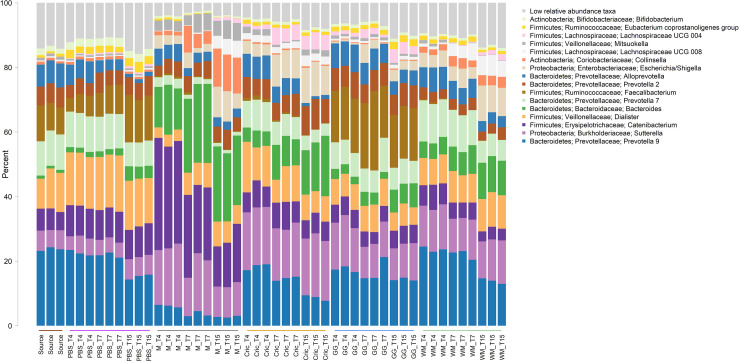
Stacked bar plot of culture community taxonomic composition of taxa at the genus level with mean relative abundance >1%. Individual culture replicates are shown. “Source” indicates starting inoculum, “PBS” indicates control cultures without added substrates, “M” indicates cultures with digested milk, “Cric” indicates cultures with digested black field cricket, “GG” represent cultures with digested grass grub, and “WM” represents cultures with digested wax moth. “T4”, T7”, and “T15” indicate 4, 7, and 15 h culture times, respectively. Low relative abundance taxa indicate sum of taxa with relative abundances <1%. Color sidebar along X-axis differentiate different substrates.

**FIGURE 3:**
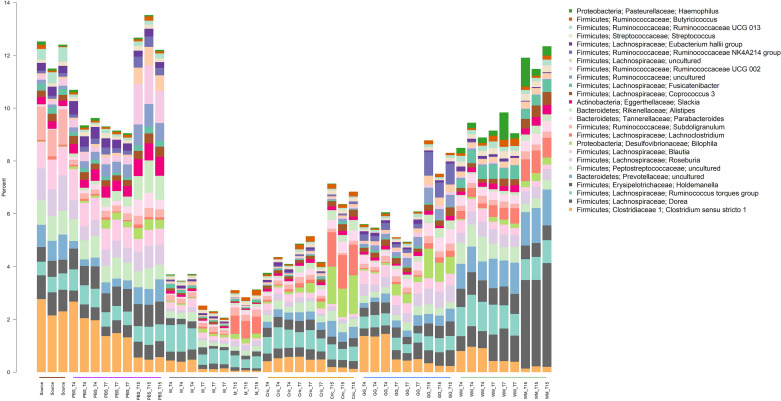
Stacked bar plot of culture community taxonomic composition of taxa at the genus level with mean relative abundances between 0.1 and 1%. Individual culture replicates are shown. “Source” indicates starting inoculum, “PBS” indicates control cultures without added substrates, “M” indicates cultures with digested milk, “Cric” indicates cultures with digested black field cricket, “GG” represent cultures with digested grass grub, and “WM” represents cultures with digested wax moth. “T4”, T7”, and “T15” indicate 4, 7, and 15 h culture times, respectively. Color sidebar along *X*-axis differentiate different substrates.

Analysis of the community taxonomic composition showed a wide range of taxa, classified to the genus level or higher, were differentially affected by the type of substrate and culture time (Figures 2, 3).

Some of the most prominent taxa that differed between substrates (including the PBS controls and milk) included the *Bacteroides*, *Prevotella*, *Sutterella*, *Catenibacterium*, *Collinsella*, *Dialister*, and *Faecalibacterium* genera. The most abundant genera (mean relative abundance > 1%) that differed significantly are shown in Table 1.

**TABLE 1:** Bacterial taxa (mean relative abundance >1%) with significantly different (FDR < 0.05) means from faecal batch cultures using different substrates across all culture times.

Phylum	Family/Genus	PBS	Milk	Black field cricket	Grass grub	Wax moth	Substrate FDR	Time (h) FDR	Interaction FDR
Bacteroidetes	*Prevotella* 9	19.84 ± 1.20^a^	4.13 ± 0.53^c^	13.86 ± 1.43^b^	16.23 ± 0.81^b^	19.86 ± 1.55^a^	<0.0001	<0.0001	0.0002
Proteobacteria	*Sutterella*	5.27 ± 0.24^d^	14.52 ± 1.38^b^	17.48 ± 0.46^a^	11.91 ± 0.74^c^	12.27 ± 0.43^c^	<0.0001	0.0001	<0.0001
Firmicutes	*Catenibacterium*	9.38 ± 0.08^b^	23.51 ± 2.68^a^	6.89 ± 0.37^bc^	5.20 ± 0.23^c^	5.14 ± 0.50^c^	<0.0001	<0.0001	<0.0001
Firmicutes	*Dialister*	15.91 ± 0.35^a^	5.79 ± 0.59^c^	10.05 ± 0.98^b^	7.33 ± 0.38^c^	9.31 ± 0.32^b^	<0.0001	0.0405	0.0001
Bacteroidetes	*Bacteroides*	2.48 ± 0.39^c^	19.8 ± 1.63^a^	9.98 ± 1.69^b^	4.35 ± 0.68^c^	7.99 ± 0.87^b^	<0.0001	<0.0001	<0.0001
Bacteroidetes	*Prevotella* 7	9.76 ± 0.55^a^	1.90 ± 0.29^c^	6.67 ± 0.72^b^	7.71 ± 0.39^b^	10.06 ± 0.85^a^	<0.0001	<0.0001	<0.0001
Firmicutes	*Faecalibacterium*	9.27 ± 1.27^b^	0.45 ± 0.07^c^	0.56 ± 0.14^c^	17.67 ± 0.97^a^	0.20 ± 0.05^c^	<0.0001	0.0144	<0.0001
Bacteroidetes	*Prevotella* 2	4.99 ± 0.28^b^	4.62 ± 0.27^b^	6.78 ± 0.52^a^	6.82 ± 0.23^a^	3.26 ± 0.13*c*	<0.0001	0.0074	0.0148
Bacteroidetes	*Alloprevotella*	3.68 ± 0.74^b^	3.5 ± 0.39^b^	4.39 ± 0.9^ab^	6.22 ± 0.54^a^	5.28 ± 0.53^ab^	<0.0001	<0.0001	<0.0001
Proteobacteria	*Escherichia*/*Shigella*	0.06 ± 0.01^c^	4.68 ± 0.97^b^	7.72 ± 1.01^a^	2.20 ± 0.63^c^	6.19 ± 0.86^ab^	<0.0001	<0.0001	0.0012
Actinobacteria	*Collinsella*	0.80 ± 0.05^bc^	6.06 ± 1.52^a^	0.43 ± 0.02^c^	0.29 ± 0.02^c^	2.62 ± 0.23^b^	<0.0001	<0.0001	<0.0001
Firmicutes	*Lachnospiraceae* UCG 008	0.39 ± 0.02^c^	1.70 ± 0.75^bc^	2.25 ± 0.15^b^	0.80 ± 0.08^c^	3.66 ± 0.76^a^	<0.0001	<0.0001	<0.0001
Firmicutes	*Mitsuokella*	0.48 ± 0.02^c^	4.39 ± 0.41^a^	1.37 ± 0.23^b^	0.81 ± 0.05^bc^	0.36 ± 0.13^c^	<0.0001	0.2887	0.0004
Firmicutes	*Lachnospiraceae* UCG 004	0.49 ± 0.05^c^	0.41 ± 0.19^c^	3.56 ± 0.62^a^	2.19 ± 0.4^b^	0.61 ± 0.24^c^	<0.0001	<0.0001	<0.0001
Firmicutes	*Eubacterium coprostanoligenes* group	2.30 ± 0.15^a^	0.29 ± 0.08^d^	0.97 ± 0.06^c^	1.62 ± 0.14^b^	1.13 ± 0.09^c^	<0.0001	0.0640	<0.0001
Actinobacteria	*Bifidobacterium*	2.45 ± 0.06^a^	0.63 ± 0.05^d^	0.88 ± 0.03^bc^	0.98 ± 0.05^b^	0.83 ± 0.04^c^	<0.0001	0.2007	0.4142

*Values indicate means ± standard error. Different superscript letters indicate significant difference in means between cultures with different substrates (Permutation ANOVA FDR < 0.05).*

Compared to PBS, digested black crickets increased the prevalence of the *Escherichia*–*Shigella* group, *Dialister* genus, and a group of unclassified *Lachnospiraceae* (FDR < 0.01). Wax moth larvae also promoted the expansion the group of unclassified *Lachnospiraceae* and *Escherichia/Shigella* (FDR < 0.01). Grass grub larvae increased the proportion of the *Prevotella* 2 group and *Faecalibacterium* compared to PBS (FDR < 0.01). The addition of milk as a substrate significantly increased the relative abundance of *Bacteroides*, *Sutterella*, *Catenibacterium*, and *Collinsella* genera, and decreased two genus level clades, recently designated as *Prevotella* 9 and 7 (Henderson et al., 2019), compared to the equivalent PBS control cultures across all time points (FDR < 0.01). Digested insect or milk substrates decreased *Bifidobacterium* proportions compared to PBS cultures (FDR < 0.01), although overall percentages of *Bifidobacterium* were low (<2.5% across all time points and substrates).

**FIGURE 4:**
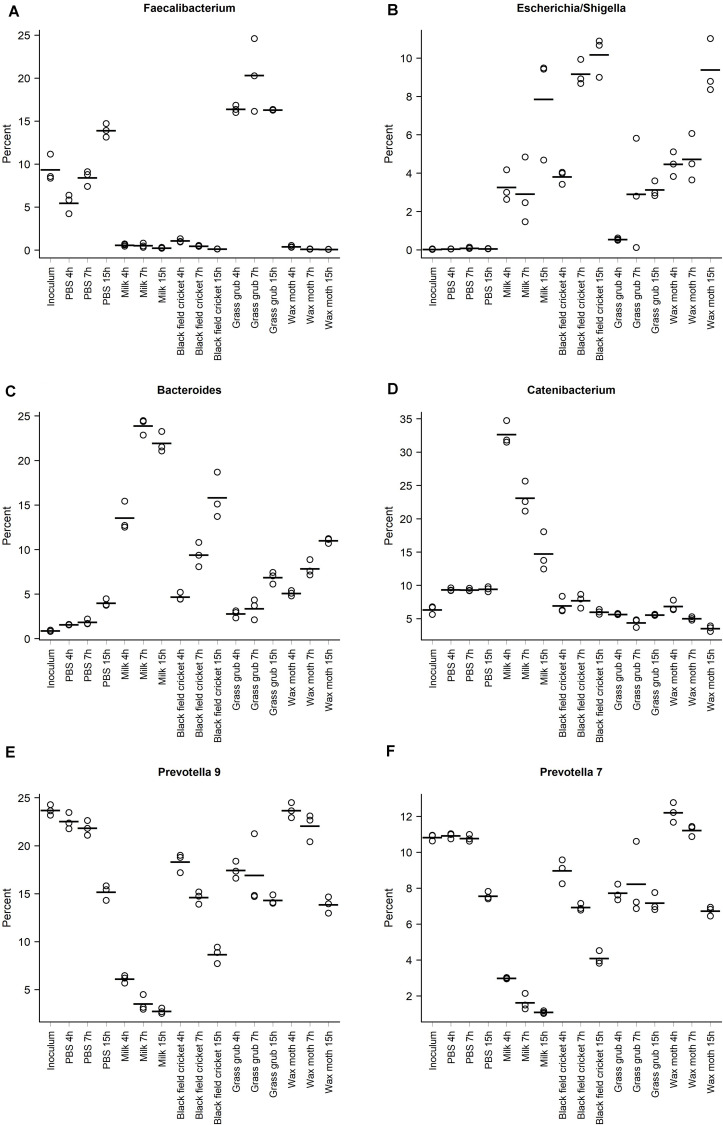
Relative abundances of the taxa with the highest overall proportions in the communities, grouped according to substrate and culture time; **(A)** Faecalibacterium, **(B)** Escherichia/Shigella, **(C)** Bacteroides, **(D)** Catenibacterium, **(E)** Prevotella 9, **(F)** Prevotella 7. Points indicate individual culture replicates except for the inoculum, where points represent technical replicates. Lines indicate treatment means.

The abundance of *Faecalibacterium* at 4 h of culture with digested grass grub was 16.4% compared to 5.5% in the PBS cultures at the same time point (Figure 4A). At 7 h, *Faecalibacterium* made up 20.3% of the community in cultures with grass grub compared to 8.4% in the equivalent PBS culture. At 15 h, both grass grub larvae and PBS cultures had similar relative abundances of *Faecalibacterium* (16.3 and 13.9%, respectively). *Faecalibacterium* proportions in all other cultures, including those with milk, were less than 1.1%.

Most taxa significantly affected by substrate type also showed differences in relative abundance over time (Tables 2–4 and Figures 4A–F). These included the *Escherichia*/*Shigella* group, which were of low abundance in the inoculum and PBS cultures (<0.1%), but on addition of all tested substrates, increased from <1% at 4 h to >10% at 15 h, depending on the substrate (Figure 4B). *Bacteroides* also increased over time in culture, with the increase especially pronounced in cultures fed with milk and black field cricket (Figure 3C). *Catenibacterium*, a genus from the *Erysipelotrichaceae* family, showed a dramatic bloom after 4 h in cultures with milk as the added substrate (>30%), but then proportions declined over time (14.7% at 15 h) (Figure 4D). *Prevotella* 9 and 7 both decreased in abundance over time in all culture treatments (Figures 4E,F).

**TABLE 2:** Bacterial taxa (mean relative abundance >1%) with significantly different (FDR < 0.05) means from fecal batch cultures using different substrates after 4 h.

Phylum	Family/Genus	PBS	Milk	Black field cricket	Grass grub	Wax moth	*P*	FDR
Bacteroidetes	*Prevotella* 9	22.53 ± 0.85^a^	6.11 ± 0.39^c^	18.31 ± 0.99^b^	17.45 ± 0.89^b^	23.67 ± 0.79^a^	<0.0001	0.0001
Proteobacteria	*Sutterella*	5.00 ± 0.67^c^	18.15 ± 1.42^a^	17.86 ± 0.06^a^	14.67 ± 1.13^b^	13.20 ± 0.67^b^	<0.0001	0.0001
Firmicutes	*Catenibacterium*	9.36 ± 0.21^b^	32.67 ± 1.78^a^	6.94 ± 1.22^c^	5.66 ± 0.09^c^	6.85 ± 0.79^c^	<0.0001	0.0002
Firmicutes	*Dialister*	16.28 ± 0.23^a^	3.53 ± 0.23^d^	12.84 ± 2.72^b^	7.67 ± 0.53^c^	8.49 ± 0.50^c^	<0.0001	<0.0001
Bacteroidetes	*Prevotella* 7	10.92 ± 0.15^b^	2.99 ± 0.04^e^	8.98 ± 0.67^c^	7.73 ± 0.45^d^	12.22 ± 0.54^a^	<0.0001	<0.0001
Bacteroidetes	*Alloprevotella*	6.29 ± 0.46^b^	5.00 ± 0.57^c^	7.12 ± 0.24^ab^	7.92 ± 0.53^a^	6.94 ± 0.67^b^	0.0003	0.0007
Bacteroidetes	*Bacteroides*	1.57 ± 0.04^c^	13.55 ± 1.64^a^	4.69 ± 0.44^b^	2.79 ± 0.42^c^	5.09 ± 0.30^b^	<0.0001	<0.0001
Bacteroidetes	*Prevotella* 2	4.28 ± 0.54^c^	4.07 ± 0.81^c^	5.89 ± 0.70^b^	7.22 ± 0.86^a^	3.19 ± 0.43^c^	0.0001	0.0004
Firmicutes	*Faecalibacterium*	5.47 ± 1.12^b^	0.59 ± 0.13^c^	1.09 ± 0.20^c^	16.39 ± 0.41^a^	0.40 ± 0.12^c^	<0.0001	0.0000
Proteobacteria	*Escherichia*/*Shigella*	0.04 ± 0.01^c^	3.27 ± 0.81^b^	3.81 ± 0.34^ab^	0.55 ± 0.06^c^	4.47 ± 0.65^a^	0.0002	0.0005
Firmicutes	*Mitsuokella*	0.46 ± 0.06^d^	3.17 ± 0.03^a^	1.91 ± 0.16^b^	0.85 ± 0.15^c^	0.87 ± 0.13^c^	<0.0001	0.0001
Firmicutes	*Eubacterium coprostanoligenes* group	2.23 ± 0.32^a^	0.60 ± 0.01^c^	1.09 ± 0.18^b^	1.37 ± 0.09^b^	1.18 ± 0.19^b^	<0.0001	0.0002
Actinobacteria	*Bifidobacterium*	2.46 ± 0.08^a^	0.80 ± 0.13^b^	0.87 ± 0.17^b^	0.96 ± 0.12^b^	0.85 ± 0.15^b^	0.0052	0.0100
Firmicutes	*Clostridium sensu stricto* 1	2.23 ± 0.39^a^	0.44 ± 0.04^d^	0.51 ± 0.09^d^	1.39 ± 0.05^b^	0.89 ± 0.08^c^	<0.0001	<0.0001
Bacteroidetes	*Prevotella* 9	22.53 ± 0.85^a^	6.11 ± 0.39^c^	18.31 ± 0.99^b^	17.45 ± 0.89^b^	23.67 ± 0.79^a^	<0.0001	0.0001

*Values indicate mean percentage ± standard deviation. Different superscript letters indicate groups that differ significantly (Permutation ANOVA FDR < 0.05).*

**TABLE 3:** Bacterial taxa (mean relative abundance >1%) with significantly different (FDR < 0.05) means from fecal batch cultures using different substrates after 7 h.

Phylum	Family/Genus	PBS	Milk	Black field cricket	Grass grub	Wax moth	*P*	FDR
Bacteroidetes	*Prevotella* 9	21.83 ± 0.78^a^	3.53 ± 0.84^c^	14.61 ± 0.65^b^	16.92 ± 3.75^b^	22.06 ± 1.44^a^	<0.0001	0.0007
Proteobacteria	*Sutterella*	4.73 ± 0.08^c^	15.65 ± 3.29^a^	15.98 ± 0.91^a^	10.42 ± 0.63^b^	11.05 ± 1.21^b^	<0.0001	0.0007
Firmicutes	*Dialister*	16.77 ± 0.58^a^	7.12 ± 0.40^c^	9.79 ± 2.33^b^	8.30 ± 0.30^bc^	9.05 ± 0.66^bc^	0.0015	0.0034
Firmicutes	*Catenibacterium*	9.33 ± 0.20^b^	23.12 ± 2.30^a^	7.71 ± 1.05^b^	4.40 ± 0.63^c^	5.01 ± 0.26^c^	<0.0001	<0.0001
Bacteroidetes	*Bacteroides*	1.86 ± 0.30^c^	23.90 ± 0.91^a^	9.41 ± 1.36^b^	3.38 ± 1.14^c^	7.86 ± 0.89^b^	<0.0001	0.0001
Bacteroidetes	*Prevotella* 7	10.78 ± 0.19^a^	1.63 ± 0.45^c^	6.93 ± 0.19^b^	8.23 ± 2.07^b^	11.22 ± 0.31^a^	<0.0001	0.0001
Firmicutes	*Faecalibacterium*	8.42 ± 0.90^b^	0.52 ± 0.26^c^	0.48 ± 0.05^c^	20.33 ± 4.23^a^	0.12 ± 0.03^c^	<0.0001	0.0001
Bacteroidetes	*Prevotella* 2	5.12 ± 0.99^b^	4.76 ± 0.52^b^	5.82 ± 0.8^ab^	6.43 ± 0.62^a^	3.39 ± 0.25^c^	0.0035	0.0072
Bacteroidetes	*Alloprevotella*	3.54 ± 0.24^c^	2.80 ± 0.31^d^	5.02 ± 0.22^b^	6.42 ± 0.42^a^	5.51 ± 0.45^b^	<0.0001	<0.0001
Proteobacteria	*Escherichia*/*Shigella*	0.09 ± 0.04^c^	2.92 ± 1.73^bc^	9.17 ± 0.66^a^	2.91 ± 2.85^bc^	4.73 ± 1.23^b^	0.0011	0.0026
Actinobacteria	*Collinsella*	0.90 ± 0.07^bc^	5.51 ± 2.37^a^	0.42 ± 0.03^c^	0.30 ± 0.07^c^	2.81 ± 0.67^b^	<0.0001	<0.0001
Firmicutes	*Lachnospiraceae* UCG 008	0.33 ± 0.05^c^	0.26 ± 0.12^c^	1.93 ± 0.20^b^	0.58 ± 0.06^c^	4.39 ± 0.85^a^	<0.0001	0.0001
Firmicutes	*Lachnospiraceae* UCG 004	0.44 ± 0.04^c^	0.05 ± 0.05^c^	4.76 ± 0.59^a^	1.87 ± 1.01^b^	0.25 ± 0.08^c^	<0.0001	<0.0001
Firmicutes	*Mitsuokella*	0.48 ± 0.08^b^	4.71 ± 1.42^a^	0.52 ± 0.18^b^	0.91 ± 0.05^b^	0.10 ± 0.05^b^	<0.0001	0.0001
Actinobacteria	*Bifidobacterium*	2.59 ± 0.27^a^	0.59 ± 0.10^c^	0.93 ± 0.03^b^	0.98 ± 0.21^b^	0.82 ± 0.13^bc^	<0.0001	0.0010
Firmicutes	*Eubacterium coprostanoligenes* group	1.95 ± 0.26^a^	0.18 ± 0.06^d^	0.91 ± 0.16^c^	1.35 ± 0.14^b^	1.37 ± 0.16^b^	<0.0003	0.0001

*Values indicate mean percentage ± standard deviation. Different superscript letters indicate groups that differ significantly (Permutation ANOVA FDR < 0.05).*

**TABLE 4:** Bacterial taxa (mean relative abundance >1%) with significantly different (FDR < 0.05) means from fecal batch cultures using different substrates after 15 h.

Phylum	Family/Genus	PBS	Milk	Black field cricket	Grass grub	Wax moth	*P*	FDR
Bacteroidetes	*Bacteroides*	4.00 ± 0.40^e^	21.94 ± 1.15^a^	15.83 ± 2.56^b^	6.87 ± 0.68^d^	11.02 ± 0.29^c^	<0.0001	<0.0001
Proteobacteria	Sutterella	6.10 ± 0.23^d^	9.76 ± 0.58^c^	18.61 ± 1.08^a^	10.65 ± 0.88^c^	12.55 ± 1.01^b^	<0.0001	<0.0001
Bacteroidetes	*Prevotella* 9	15.17 ± 0.78^a^	2.76 ± 0.29^d^	8.66 ± 0.88^c^	14.32 ± 0.49^ab^	13.85 ± 0.85^b^	<0.0001	<0.0001
Firmicutes	*Dialister*	14.68 ± 0.63^a^	6.71 ± 0.95^cd^	7.52 ± 0.73^c^	6.00 ± 0.81^d^	10.39 ± 0.36^b^	<0.0001	<0.0001
Firmicutes	*Catenibacterium*	9.44 ± 0.37^b^	14.75 ± 2.92^a^	6.01 ± 0.36^c^	5.56 ± 0.1^cd^	3.54 ± 0.43^d^	<0.0001	<0.0001
Firmicutes	*Faecalibacterium*	13.91 ± 0.78^b^	0.24 ± 0.06^c^	0.13 ± 0.01^c^	16.31 ± 0.04^a^	0.09 ± 0.02^c^	<0.0001	<0.0001
Proteobacteria	*Escherichia*/*Shigella*	0.06 ± 0.01^c^	7.86 ± 2.75^a^	10.18 ± 1.04^a^	3.13 ± 0.41^b^	9.39 ± 1.42^a^	<0.0001	<0.0001
Bacteroidetes	*Prevotella* 2	5.59 ± 0.40^bc^	5.03 ± 0.95^c^	8.62 ± 1.03^a^	6.81 ± 0.49^b^	3.21 ± 0.55^d^	<0.0001	<0.0001
Bacteroidetes	*Prevotella* 7	7.57 ± 0.22^a^	1.09 ± 0.07^d^	4.09 ± 0.37^c^	7.18 ± 0.51*a*^b^	6.73 ± 0.26^b^	<0.0001	<0.0001
Actinobacteria	*Collinsella*	0.63 ± 0.02^c^	11.41 ± 0.36^a^	0.40 ± 0.09^c^	0.32 ± 0.02^c^	3.18 ± 0.23^b^	<0.0001	<0.0001
Firmicutes	*Lachnospiraceae* UCG 008	0.45 ± 0.06^d^	4.68 ± 0.65^b^	2.70 ± 0.57^c^	1.08 ± 0.13^d^	5.76 ± 0.78^a^	<0.0001	<0.0001
Bacteroidetes	*Alloprevotella*	1.20 ± 0.13^d^	2.71 ± 0.17^c^	1.03 ± 0.21^d^	4.31 ± 0.28^a^	3.40 ± 0.13^b^	<0.0001	<0.0001
Firmicutes	*Lachnospiraceae* UCG 004	0.67 ± 0.08^c^	1.17 ± 0.26^c^	4.77 ± 0.54^a^	3.47 ± 0.89^b^	1.49 ± 0.50^c^	<0.0001	<0.0001
Firmicutes	*Mitsuokella*	0.50 ± 0.06^c^	5.28 ± 0.59^a^	1.69 ± 0.36^b^	0.68 ± 0.11^c^	0.12 ± 0.02^c^	<0.0001	<0.0001
Firmicutes	*Eubacterium coprostanoligenes* group	2.71 ± 0.46^a^	0.07 ± 0.01^d^	0.90 ± 0.15^c^	2.16 ± 0.12^b^	0.84 ± 0.10^c^	<0.0001	<0.0001
Actinobacteria	*Bifidobacterium*	2.32 ± 0.13^a^	0.51 ± 0.03^d^	0.84 ± 0.08^bc^	1.00 ± 0.11^b^	0.82 ± 0.10^c^	<0.0001	<0.0001
Firmicutes	*Dorea*	0.58 ± 0.02^b^	0.10 ± 0.03^c^	0.29 ± 0.01^bc^	0.53 ± 0.06^b^	3.52 ± 0.36^a^	<0.0001	<0.0001

*Values indicate mean percentage ± standard deviation. Different superscript letters indicate groups that differ significantly (Permutation ANOVA FDR < 0.05).*

**FIGURE 5:**
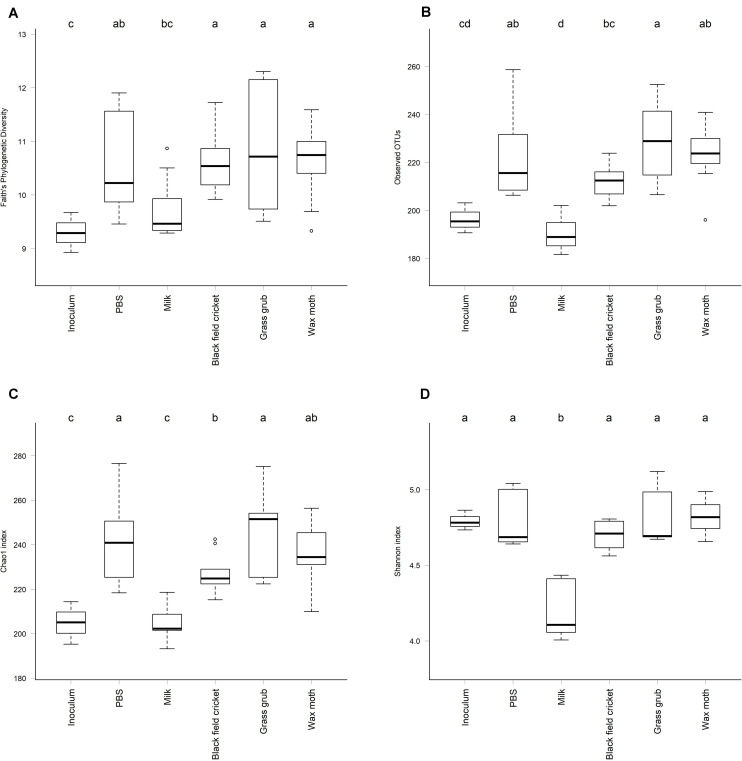
Alpha diversity of cultures at a sequence sampling depth of 13,500 reads; **(A)** Faith’s Phylogenetic Diversity, **(B)** Observed OTUs, **(C)** Chao1 index, **(D)** Shannon index. All culture time points aggregated, as alpha diversity did not differ significantly between culture 4, 7, or 15 h (ANOVA *P* > 0.08). Cultures that differed significantly in diversity are labeled with different letters (ANOVA *P* < 0.05).

Indicators of alpha diversity, as measured by Faith’s Phylogenetic Diversity, observed OTUs, and chao1 and Shannon indices, increased in all cultures compared to the inoculum except where milk was the added substrate (*P* < 0.01) (Figure 5). Alpha diversity did not differ significantly between culture time points for any of the indices measured (*P* > 0.08).

## Discussion

Our results clearly show that different insects have the potential to modify the human gut microbiome, at least *in vitro*. While the effects *in vivo* with New Zealand insects have yet to be demonstrated, a previous study by Stull et al. (2018), showed consuming cricket flour can have a beneficial effect on the human microbiome. In that report, only minor changes were observed, whereas our study showed the different digested insects, at least *in vitro*, have the potential to induce substantial changes in the microbiome structure, with each insect having distinct effects. However, the large inter-personal variation in the general human population would likely act to obscure dietary effects when compared to results from our *in vitro* study. The difference in magnitude of effect observed *in vitro* and *in vivo* is also likely to be influenced by dose; in the clinical study referenced above, the cricket flour contributed a relatively minor proportion of the overall diet for each participant, whereas in our *in vitro* study, the digested insects constituted a relatively larger component of the available substrate for the microbiome. Indeed, in a study examining the effects of an insect diet in laying hens, dramatic changes were observed in the caecal microbiome when insects were the sole dietary source (Borrelli et al., 2017).

The increased *Faecalibacterium* observed in the cultures with digested grass grub larvae is a particularly interesting finding due to the association of this bacterium with health promoting properties (Sokol et al., 2008; Ulluwishewa et al., 2015; Martín et al., 2017). *Faecalibacterium* is reduced in Crohn’s disease patients (Sokol et al., 2009) and cellular and animal studies have shown this bacterium can exert anti-inflammatory effects (Sokol et al., 2008; Ulluwishewa et al., 2015). The rapid expansion in *Faecalibacterium* after only 4 h of incubation with digested grass grub larvae suggests the presence of substrates that can be rapidly utilized by this bacterium. At 15 h, when *Faecalibacterium* proportions in the grass grub and PBS cultures became similar, it is possible that these *Faecalibacterium* accessible substrates were depleted by then. Furthermore, the relatively high proportions of *Faecalibacterium* observed in both grass grub and PBS cultures at 15 h might be explained by the presence of endogenous substrates in the inoculum. However, the absence of *Faecalibacterium* in cultures with the other substrates would suggest these substrates led to other bacteria having a competitive advantage over *Faecalibacterium*, which remained for the duration of the incubation time. Although the specific compound in grass grub that could account for the comparative increase in *Faecalibacterium* remains unknown at this stage, *Faecalibacterium* are well known as users of host-derived substrates such N-acetylglucosamine (Lopez-Siles et al., 2012; Heinken et al., 2014), which is the building block of chitin. It may be speculated that the *in vitro* digestion process caused the N-acetylglucosamine from the grass grub larvae to be more accessible compared to the other types of insects or that the chitin content of grass grubs is higher than that of the other insects.

Our study also highlighted the different effect of insects on the microbiome compared to milk, which was chosen as a reasonable representative of a widely consumed type of “whole” food. Some of these differences include a large decrease in *Prevotella* in the cultures with digested milk compared to PBS. In cultures with digested insects, *Prevotella* relative abundances at the 4 h timepoint were similar to the PBS cultures, but these too fell as culture time progressed. *Prevotella* are often associated with diets high in plant material and dietary fiber (De Filippo et al., 2010; Makki et al., 2018), and as such, microbiomes dominated by *Prevotella* are generally thought of as beneficial (Makki et al., 2018). There is also some evidence that people with microbiomes characterized by high *Prevotella* to *Bacteroides* ratios lose more body weight on diets high in fiber compared to subjects with lower ratios (Hjorth et al., 2018). Like *Faecalibacterium*, *Prevotella* are also prominent users of host-derived substrates, including mucin (Wright et al., 2000), which contain sulfate conjugated N-acetylglucosamine, and cultured representatives of *Prevotella* have been shown to utilse N-acetylglucosamine as a sole substrate (Avgustin et al., 1997).

The distinctive effects of each type of insect is likely to be influenced by their biology and genetic differences that lead to different structural and chemical compositions. However, environmental factors can also not be ruled out. For example, it is conceivable that the effects of each type of insect may be influenced by their diet. For example, the grass grubs from the wild may have contained soil in their gut, ingested while feeding on roots and organic matter in pasture. Any non-digestible organic material remaining in the insect gut could have formed part of the substrate during the fecal fermentation. This concept also raises the possibility of modifying the effects of edible insects on the human gut microbiome by altering their forage material. In this way, novel bioactives from native plants not otherwise suitable for human consumption could be leveraged to make novel functional foods and ingredients. Finally, the resident microbes in the insect gut itself could contribute to the host microbial community.

The relative abundances of *Escherichia*/*Shigella* also differed greatly between the type of substrates added, with the largest increases seen with crickets and wax moths as substrates after 15 h. Interestingly, like *Faecalibacterium* and *Prevotella*, *Escherichia* are also able to utilize N-acetylglucosamine and related compounds such as sialic acid (Plumbridge and Vimr, 1999; Álvarez-Añorve et al., 2005). It is also possible that the increase in *Escherichia*/*Shigella* after the addition of substrates (milk or insects) reflects the unintended introduction of oxygen into the cultures. However, the increase in *Faecalibacterium* from adding grass grub suggests otherwise as *Faecalibacterium* is extremely oxygen sensitive (Duncan et al., 2002). Although the expansion of *Escherichia*–*Shigella* and *Sutterella* in cultures with digested insects may warrant caution due to their associations with some diseases or disorders (Garrett et al., 2010; Byndloss et al., 2017), many Proteobacteria are commensals and not associated with dysbiosis (Moon et al., 2018).

The relative abundance of *Collinsella* was increased in cultures with bovine milk, and this observation matched *in vivo* results observed in an animal study (Rettedal et al., 2019). We also observed increased proportions *Catenibacterium* in cultures with milk as a substrate, which align with results from a recent study where consumption of a fermented dairy product also increased *Catenibacterium* abundance in human volunteers (Volokh et al., 2019). *Bacteroides* were also increased in the cultures with milk over time, as they were with all insect substrates. *Bacteroides* are well known as “generalists” (Louis, 2017), capable of using a range of substrates, including mucins and N-acetylglucosamine subunits (Chen et al., 2002; Tailford et al., 2015), which may explain their ability to grow with all of the substrates we studied.

Although a chemical analysis of the insect retentate following digestion was not carried out, it is reasonable to assume that a substantial proportion of the material influencing the microbiome was chitin, as this is known to resist digestion (Muzzarelli et al., 2012), despite the presence of a low level of chitinase gene expression in the human stomach (Ohno et al., 2013). In particular, the taxa that seemed to increase the most in response to the insect substrates were those with representatives that are known to utilize N-acetylglucosamine, the major building block of chitin. However, the specific responses to each insect substrate varied between the taxa. Although a comparative chemical analysis of the chitin composition and structure between the insects in our study has not been carried out to the best of our knowledge, it is known that chitin can carry modifications such as acetylation. The degree of acetylation in chitin varies (Davies and Hayes, 1988) and this can influence its mechanical properties (Cui et al., 2016), which may also help explain the different effect each insect had as a substrate. However, the influence of other components that also escaped digestion on the microbiome cannot be ruled out.

While no presently available *in vitro* culture system can truly replicate *in vivo* conditions, the digestion and fermentation model we used represents a reasonable approximation of conditions *in vivo*: the digestion protocol used followed a standardized method developed through international consensus (Minekus et al., 2014) and the addition of a dialysis step following digestion results in a substrate that better approximates the substrates which would reach the large intestine. The similarity of our PBS cultures compared to the starting inoculum provides confidence that the culture conditions used in our study are a reasonable approximation of the activity of the *in vivo* microbiome.

Our study shows New Zealand insects may have the potential to differentially modify the human gut microbiome, with the increase in *Faecalibacterium* from adding digested grass grub meriting further investigation. Given the increasing consumer interest in alternatives to foods from traditional livestock, continued study of novel additional food sources such as New Zealand edible insects are warranted. It must be noted that while our results raise the possibility for differential modulation of the human microbiome based on the type of insect consumed, the limitations of our study, such as the low sample size and translatability of *in vitro* models, means further studies are required to validate our findings.

## Data Availability Statement

The datasets generated for this study can be found in the NCBI Sequence Read Archive accession PRJNA566047.

## Ethics Statement

All fecal donors gave written informed consent for the use of their fecal material in this study. Health and Disabilities Committees (HDEC) approval was not required for this study, in accordance to their guidelines, as no personal identifiable information was collected and sample collection was only minimally invasive.

## Author Contributions

WY designed the study. WY, MM, and PP wrote the manuscript. WY and ER performed the experiments. WY, SA, and ER analyzed and interpreted the data. MM collected grass grub larvae and black field crickets from the wild. All authors contributed to the study design, manuscript discussion and revision, and approved the final version.

## Conflict of Interest

The authors declare that the research was conducted in the absence of any commercial or financial relationships that could be construed as a potential conflict of interest.

## Footnotes

1 http://biosuppliers.nz
